# Genetic factors influencing milk and fat yields in tropically adapted dairy cattle: insights from quantitative trait loci analysis and gene associations

**DOI:** 10.5713/ab.23.0246

**Published:** 2023-11-01

**Authors:** Thawee Laodim, Skorn Koonawootrittriron, Mauricio A. Elzo, Thanathip Suwanasopee, Danai Jattawa, Mattaneeya Sarakul

**Affiliations:** 1 Department of Animal Science, Faculty of Agriculture at Kamphaeng Saen, Kasetsart University Kamphaeng Saen Campus, Nakhon Pathom, 73140, Thailand; 2 Tropical Animal Genetic Special Research Unit (TAGU), Kasetsart University, Bangkok, 10900, Thailand; 3 Department of Animal Science, Faculty of Agriculture, Kasetsart University, Bangkok, 10900, Thailand; 4 Department of Animal Sciences, University of Florida, Gainesville, 32611-0910, FL, USA; 5 Department of Animal Science, Faculty of Agriculture and Technology, Nakhon Phanom University, Nakhon Phanom, 48000, Thailand

**Keywords:** Adaptation, Dairy Cattle, Fat Yield, Milk Yield, Gene Associations, Tropics

## Abstract

**Objective:**

The objective of this study was to identify genes associated with 305-day milk yield (MY) and fat yield (FY) that also influence the adaptability of the Thai multibreed dairy cattle population to tropical conditions.

**Methods:**

A total of 75,776 imputed and actual single nucleotide polymorphisms (SNPs) from 2,661 animals were used to identify genomic regions associated with MY and FY using the single-step genomic best linear unbiased predictions. Fixed effects included herd-year-season, breed regression, heterosis regression and calving age regression effects. Random effects were animal additive genetic and residual. Individual SNPs with a p-value smaller than 0.05 were selected for gene mapping, function analysis, and quantitative trait loci (QTL) annotation analysis.

**Results:**

A substantial number of QTLs associated with MY (9,334) and FY (8,977) were identified by integrating SNP genotypes and QTL annotations. Notably, we discovered 17 annotated QTLs within the health and exterior QTL classes, corresponding to nine unique genes. Among these genes, Rho GTPase activating protein 15 (*ARHGAP15*) and catenin alpha 2 (*CTNNA2*) have previously been linked to physiological traits associated with tropical adaptation in various cattle breeds. Interestingly, these two genes also showed signs of positive selection, indicating their potential role in conferring tolerance to trypanosomiasis, a prevalent tropical disease.

**Conclusion:**

Our findings provide valuable insights into the genetic basis of MY and FY in the Thai multibreed dairy cattle population, shedding light on the underlying mechanisms of tropical adaptation. The identified genes represent promising targets for future breeding strategies aimed at improving milk and fat production while ensuring resilience to tropical challenges. This study significantly contributes to our understanding of the genetic factors influencing milk production and adaptability in dairy cattle, facilitating the development of sustainable genetic selection strategies and breeding programs in tropical environments.

## INTRODUCTION

Dairy farming in tropical and subtropical regions places importance not only on milk yield (MY) and quality but also on the environmental adaptation of dairy cattle. Environmental adaptation, including disease and parasite resistance, as well as heat tolerance, plays a crucial role in reducing animal stress and promoting environmentally friendly production practices [1]. While taurine breeds have shown high productive efficiency under temperate conditions, indicine cattle, known for their adaptability, have demonstrated resilience under poor input conditions and resistance to tropical diseases [2]. Thus, a breeding strategy involving *Bos indicus* - *Bos taurus* crosses has been employed in tropical and subtropical regions to enhance thermal and parasite tolerance while retaining favorable traits of *Bos taurus* cattle [3].

The Thai multibreed dairy cattle population is the result of an upgrading strategy involving crossbreeding of multiple *Bos taurus* and *Bos indicus* breeds with Holstein to obtain animals with high MY and adaptability to tropical conditions [4]. The majority of dairy cows in this population are crossbred animals with over 75% Holstein (91%) and the remainder comes from various *Bos indicus* (Red Sindhi, Sahiwal, Brahman, and Thai Native) and *Bos taurus* (Brown Swiss, Red Danish, and Jersey) breeds. Moreover, an animal could have as many as eight different cattle breeds represented in it [4,5]. Previous genome-wide association studies (GWAS) in the Thai population identified sets of putative single nucleotide polymorphisms (SNPs) associated with MY, fat yield (FY), and age at first calving that differ slightly from those found in *Bos taurus* breeds in temperate regions [6]. These SNP markers are believed to be associated with milk production in Thai multibreed dairy cattle either directly or indirectly by influencing their adaptability to high heat and humidity, thus allowing them to fully express their genetic potential under tropical conditions [7].

Thus, the objective of this study was to identify a set of genes associated with 305-day MY and FY that is also involved in the adaptability of the Thai multibreed dairy cattle population to tropical conditions. Understanding the genetic basis of these traits and their relationship to adaptability will provide valuable insights for improving dairy production in tropical and subtropical regions.

## MATERIALS AND METHODS

### Animals and phenotypes

The dataset used in this study was obtained from commercial dairy farms adhering to Good Agricultural Practices and Good Farming Management Practices mandated by the relevant authorities. Ethical clearance (ACKU60-AGR-009) was granted by the Institutional Animal Care and Use Committee of Kasetsart University, ensuring animal welfare and ethical treatment. A total of 8,361 first-lactation cows with their first calving between 1989 and 2014 were included in the study. These cows had complete pedigree and phenotypic information. Characteristics and farm management of all the animals were previously described in Laodim et al [7]. All phenotypic records were collected at 810 farms located in the Northern, Northeastern, Central, Western, and Southern regions of Thailand. Milk yield and FY phenotypes were gathered monthly from individual cows. These monthly test-day milk and fat records were used to calculate 305-d MY (kg) and 305-d FY (kg) using a test-interval procedure [8,9]. The average MY and FY in this population were 4,311.25± 1,116.68 kg and 153.92±50.50 kg, respectively.

### Genotype data

Tissue sample collection included semen from 89 sires and blood from the jugular vein of 2,572 dams. Genomic DNA from semen was extracted with the GenElute Mammalian Genomic DNA Miniprep Kit (Sigma, Ronkonkoma, NY, USA), whereas the MasterPure DNA Purification kit for blood version II (EPICENTRE Biotechnologies, Madison, WI, USA) was used to obtain genomic DNA from blood samples. Animals were genotyped with GeneSeek Genomic Profiler (GGP) chips (9K, 20K, 26K, or 80K). Imputation to the GGP80K chip was performed with FImpute version 2.2 [10], utilizing a reference population of 139 animals genotyped with GGP80K. The accuracy of imputation with program FImpute was 93.94% [11].

Quality control procedures were implemented using PLINK software version 1.7 (http://pngu.mgh.harvard.edu/purcell/plink/). SNPs were required to have a minimum minor allele frequency of 0.01 and a minimum call rate of 0.90 to be included in the study. The edited genotype file contained a total of 75,776 SNP markers distributed across the 29 autosomes and the X chromosome. This quality control process yielded high-quality genotypic data, ensuring the reliability and accuracy of subsequent analyses in our study.

### Genome-wide association analysis

A GWAS was conducted using the single-step genomic best linear unbiased prediction (ssGBLUP) approach [12]. The two-trait genomic-polygenic model implemented for ssGBLUP can be represented as follows:


y=Xb+Za+e

where ***y*** is the vector of phenotypic records for MY and FY. Vector ***b*** is the vector of fixed effects, which includes contemporary group (herd-year-season subclasses), breed regression (linear function of the expected other fraction in each animal), heterosis regression (linear function of heterozygosity), and calving age regression effects. Contemporary groups were defined as groups of cows calving in the same herd, year, and season. Calving seasons were defined as winter (November to February), summer (March to June), and rainy (July to October). There were 3,848 contemporary groups with an average of 2.18 cows per contemporary group. Heterozygosity was calculated as the product of the expected Holstein fraction of the sire and the expected Other breed fraction of the dam plus the expected Other breed fraction of the sire times the expected Holstein fraction of the dam [5].

Random vector ***a*** contains additive genetic effects. Vector ***a*** follows a normal distribution with $a\sim N(0,H\sigma_{a}^{2})$, where ***H*** represents the genomic-polygenic relationship matrix constructed according to Aguilar et al [12], and $\sigma_{a}^{2}$ represents the additive genetic variance. Random vector ***e*** is the vector of residuals. Vector ***e*** has a normal distribution $e\sim N(0,I\sigma_{e}^{2})$, where ***I*** denotes the identity matrix and $\sigma_{e}^{2}$ is the residual variance. Incidence matrices ***X*** and ***Z*** relate phenotypic records to fixed effects in vector ***b*** and random effects in vector ***a***, respectively. The inverse of the ***H*** matrix (***H****^−1^*) was defined as follows:


$$H^{-1}=A^{-1}+\left[\begin{array}{cc} 0 & 0\\ 0 & G^{-1}-A_{22}^{-1} \end{array}\right],$$

where ***A****^−1^* is the inverse of the relationship matrix based on pedigree information; ***G****^−1^* is the inverse of the genomic relationship matrix; $A_{22}^{-1}$ is the inverse of the matrix of additive genetic relationships between genotyped animals. The ***G*** matrix was built using the methods proposed by VanRaden [13], as follows:


$$G=\frac{ZZ^{\prime }}{2\;\Sigma p_{j}(1-p_{j})}$$

where ***p****_j_* is the frequency of the ***B*** allele in locus j and ***Z*** is an incidence matrix of SNP effects whose elements are equal to (0–2p_j_) if the genotype for locus j is equal to AA, (1–2p_j_) if the genotype for locus j is equal to AB or BA, and (2–2p_j_) if the genotype for locus j is equal to BB [13].

Restricted maximum likelihood variance and covariance components for ssGBLUP were computed using an average information algorithm with program AIREMLF90 [14]. These variance components were used to compute genomic estimated breeding values (GEBVs) with program BLUPF90 [15]. The GEBV were computed using program POSTGSF90 from the BLUPF90 Family of Programs. Program POSTGSF90 was also used to estimate SNP effects, p-values, and the proportion of the additive genetic variance explained by SNPs for MY and FY. The SNP effects were predicted utilizing the following equation:


$$\hat{u}=IZ^{\prime }{\left({\mathrm{ZIZ}}^{\prime }\right)}^{-1}\hat{a},$$

where ***û*** is a vector of predicted SNP values; ***I*** is an identity matrix; ***Z*** is a matrix relating individual animals to their phenotypes; and ***â*** is a vector of GEBV for genotyped animals. The percentage of the additive genetic variance explained by individual SNP marker was calculated as the ratio of additive genetic variance accounted for individual SNP divided by the total additive genetic variance [16]. The p-value of SNP markers were generated by back solving for SNP values from the GEBV. The p-value for the ith SNP was calculated as $2\left[1-\Phi \;\left(\left|\frac{{\hat{u}}_{i}}{\mathrm{sep}({\hat{u}}_{i})}\right|\right)\right]$, where **Φ** is the cumulative standard normal distribution and **sep(û_i_)** is the square root of the predicted error variance for the ith SNP. Subsequently, Manhattan plots were generated to visualize the additive genetic variance explained by individual SNP and their corresponding p-values for MY and FY. The Manhattan plot was utilized for plotting SNP additive genetic variances, whereas the "CMplot" R package [17] was employed for visualization of the p-values and the Q-Q plots.

Two thresholds were applied to the identification of significant SNPs associated with MY and FY. Firstly, SNP with a false discovery rate greater than 0.1 (i.e., greater than −log_10_(p-value) = −log_10_(0.1/75,776) = 5.87) were considered to exhibit suggestive associations. Secondly, all SNP markers with a −log(p-value) greater than or equal to 4 were regarded as putative regions associated with MY and FY. These two thresholds enabled the identification of a limited number of significant SNPs, facilitating the investigation of potential biological functions of genomic regions associated with MY and FY. Subsequently, SNPs with a p-value smaller than 0.05 were selected for gene mapping, function analysis, and quantitative trait loci (QTL) annotation analysis.

### Gene identification and function analysis

Positional genes for MY and FY were identified by considering a genomic region on each side of significant SNPs with normal p-value of <0.05 for each trait (4,056 SNPs for MY and 3,698 SNPs for FY). This genomic region went from 15 kb upstream to 15kb downstream of the location of the significant SNPs in the reference bovine genome assembly (ARS-UCD 1.2) embedded in the Ensembl Genome Browser (https://www.ensembl.org/index.html). The variant annotation category was based on Ensembl variant effect predictors (VEP) [18]. The VEP annotations for the variants are shown in Supplementary Figure S1 for MY and Supplementary Figure S2 for FY. Numbers of genes per chromosome for MY and FY identified by SNP genotypes inside or within 15 kb upstream and 15 kb downstream of genes in the Ensembl *Bos taurus* genome assembly are shown in Supplementary Table S1. The total number of genes associated with MY was 1,951 and 1,684 for FY.

The gene ontology (GO) annotation and Kyoto encyclopedia of genes and genomes (KEGG) pathway enrichment analyses were performed separately for each trait using the 2021 version of the Database for Annotation, Visualization, and Integrated Discovery (DAVID) software (https://david.ncifcrf.gov/). The *Bos taurus* gene list of DAVID was used as the reference genome. The DAVID software offers multiple biological descriptors (GO terms: biological process (BP), molecular function (MF) and cellular component [CC]) and KEGG pathways for genes based on the characteristics of encoded proteins. The Benjamini-Hochberg correction was applied to search for overrepresentation of significantly associated genes among all genes in a given GO term and KEGG pathway. Finally, only GO terms and KEGG pathways with a p-value <0.05 were considered statistically significant and used for further interpretation.

### Quantitative trait loci annotation analysis

A list of SNPs associated with MY and FY was used for QTL annotation with the R package Genomic Annotation in Livestock for Positional Candidate Loci (GALLO) (https://github.com/pablobio/GALLO; [19]). The QTL were annotated using a .gff file from the Animal QTL database containing bovine information from the ARS-UCD1.2 bovine genome assembly.

## RESULTS AND DISCUSSION

### Genome-wide association analysis

The additive genetic variance explained by each of the 75,776 SNP across the entire genome was visualized using a Manhattan plot (Supplementary Figure S3). The proportion of the additive genetic variance explained by individual SNPs ranged from 0.0247×10^−10^ to 0.0419 for MY, and from 0.0011 ×10^−10^ to 0.0373 for FY. The small magnitude of the percentages of the additive genetic variance contributed by all SNPs indicated that MY and FY were influenced by a multitude of SNPs across the genome, each contributing with a small fraction of the total additive genetic variance.

**Figure 1:**
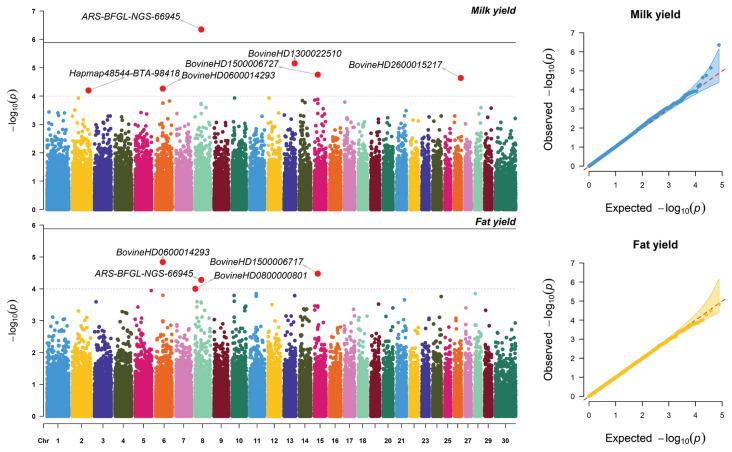
Manhattan and Q-Q plots based on GWAS results for milk yield and fat yield. Suggestive SNP associations are those with −log_10_(p) values above 5.87, and suggestive putative regions associations require −log_10_(p) values equal to or greater than 4. GWAS, genome-wide association studies; SNP, single nucleotide polymorphisms.

Significant SNPs associated with MY and FY are shown in Table 1. The Manhattan and Q-Q plots highlighting these associations are presented in Figure 1. The Q-Q plots showed that there was no apparent systematic deviation with most of the points distributed around the diagonal. The GWAS revealed only one significantly associated SNP at −log (p-value)≥5.87 for MY, while no significant SNPs associated with FY existed for this criterion. This significant SNP (ARS-BFGL-NGS-66945) was intergenic but more than 15 kb from the nearest gene (Table 1). The additive genetic variance explained by ARS-BFGL-NGS-66945 was 0.0419%. However, there were five significant SNPs associated with MY showing a −log(p-value) ≥4. These SNPs were in chromosomes 2 (Hapmap48544-BTA-98418), 6 (BovineHD 0600014293), 13 (BovineHD1300022510), 15 (BovineHD 1500006727), and 26 (BovineHD2600015217). The additive genetic variances explained by these SNPs ranged from 0.0259 to 0.0338. Most of the significant SNPs resided within genes, including F-box protein 36 (*FBXO36*), protein prenyltransferase alpha subunit repeat containing 1 (*PTAR1*), beta-1,4-galactosyltransferase 5 (*B4GALT5*), and protein tyrosine phosphatase receptor type E (*PTPRE*) (Table 1).

There were four significant SNPs (−log(p-value) ≥4) associated with FY, namely BovineHD0600014293 in chromosome 6, BovineHD0800000801 and ARS-BFGL-NGS-66945 in chromosome 8, and BovineHD1500006717 in chromosome 15 (Table 1; Figure 1). The additive genetic variance explained by each of these SNPs ranged from 0.0043% to 0.0373%. Only one of these SNP (ARS-BFGL-NGS-66945) was found within a known gene, namely *PTAR1*. Notably, the *PTAR1* gene was associated with both MY and FY in this population. However, the set of significant SNPs for MY and FY here differed from those found in the Thai multibreed dairy population with 8,096 SNPs [6] and 50,908 SNPs [20]. This finding agreed with previous reports indicating that different GWAS models and different number of SNPs or number of genotyped animals would yield different sets of significant SNPs for the traits of interest [21–23].

These findings highlight the potential significance of the *PTAR1* gene in affecting MY and FY under tropical conditions. The identification of this gene as a common factor influencing these two traits suggests its potential role in regulating milk and fat synthesis. Further research into the biological functions and mechanisms of action of *PTAR1* could provide valuable insights into the genetic control of MY and FY, and potentially contribute to the development of targeted breeding strategies for enhanced MY and FY in the Thai multibreed dairy cattle population.

The *FBXO36*, *B4GALT5*, and *PTPRE* genes were found to be associated with MY and FY in this population. Interestingly, these genes have also been implicated in various traits in other cattle populations, indicating their potential multifunctional roles. While some of these associations involved biological functions related to adaptation traits, others were linked to specific diseases and infections [24–32].

*FBXO36*, a member of the F-box protein family, is known to be involved in protein ubiquitination and plays a critical role in various cellular functions, including the cell cycle, circadian clocks, nutrient sensing, and signal transduction. It has also been associated with bovine mastitis resistance in Holstein [24]. Bovine mastitis is a prevalent inflammation of the mammary gland caused by physical trauma or microbial infections. This disease significantly impacts the dairy industry due to reduced MY and compromised milk quality. Furthermore, *FBXO36* has shown associations with differences in clinical scores between bovine respiratory syncytial virus (BRSV)-challenged and control groups in Holstein-Friesian [25]. BRSV is a major pathogen causing bovine respiratory disease, which poses significant economic challenges to dairy cattle production.

These findings underscore the potential relevance of *FBXO36* for disease resistance and adaptation to tropical conditions in dairy cattle. The association of this gene with MY and FY in the Thai multibreed population suggests its involvement in regulating milk and fat synthesis. Additionally, the presence of *FBXO36* in other cattle populations and its role in disease resistance highlights its significance in a broader context.

Genes *B4GALT5* and *PTPRE* were associated with MY and FY in the Thai multibreed population. The *B4GALT5* gene belongs to the β-1,4-galactosyltransferase (*B4GALT*) family. It is known to play a role in embryonic development as well as in immune and inflammatory responses. Gene *B4GALT5* was reported to be a candidate gene associated with mastitis resistance caused by *Escherichia coli* and *Streptococcus uberis* in dairy cows [26]. In addition, *B4GALT5* was associated with innate immune response to *Mycoplasma bovis* infection in Holstein cows [27]. *Mycoplasma bovis* is a known cause of mastitis leading to decreased milk production and significant economic losses. Further, *B4GALT5* was found to be expressed in bovine mammary epithelial cells and involved in upregulating the immune system during bovine leukemia virus infection, resulting in a decrease in the incidence of mastitis [28].

The *PTPRE* gene belongs to the protein tyrosine phosphatase (PTP) family and plays a crucial role in regulating various cellular processes, including cell growth, differentiation, the mitotic cycle, and oncogenic transformation (https://www.genecards.org/cgi-bin/carddisp.pl?gene=PTPRE). Zhang et al [29] indicated that the *PTPRE* gene inhibited the activation of the *KIT* gene. The *KIT* gene is known to be involved in hematopoiesis, gametogenesis, and melanogenesis and has been reported to be a candidate gene for spotting loci in various cattle breeds including Italian Holstein-Friesian, Italian Brown, Italian Simmental, Jersey, Rendena, Reggiana, Modenese, Angus, and Hereford [30,31]. Furthermore, *PTPRE* has been identified as a candidate gene for residual feed intake in dairy cattle [32].

These findings highlight the potential roles of *B4GALT5* and *PTPRE* in various BPs and their associations with economically important traits in cattle. The involvement of *B4GALT5* in immune response and mastitis resistance underscores its significance in maintaining mammary health and milk production. It is important to note that adaptation to tropical climates encompass a wide range of morphological, behavioral, and physiological traits including light hair color, reduced feed intake, and disease resistance [1]. The associations observed in this study provide additional evidence of the complex genetic architecture underlying dairy production traits while contributing to the development of improved selection strategies for increasing MY and FY under tropical conditions.

### Functional enrichment analysis

**Figure 2:**
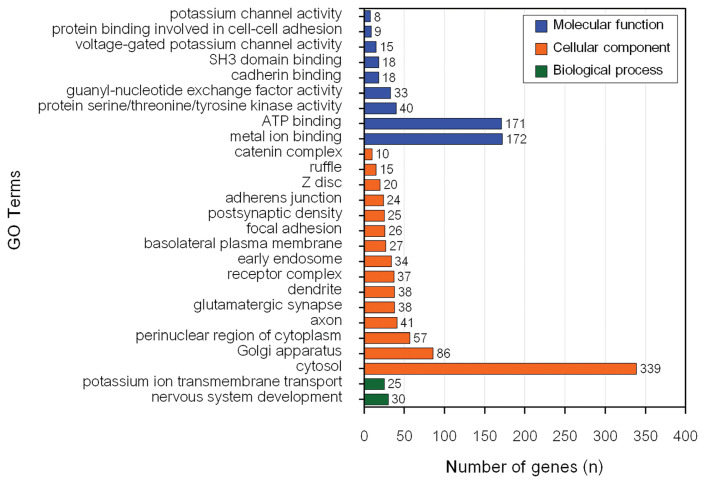
Functional terms significantly enriched corresponding to genes associated with milk yield.

**Figure 3:**
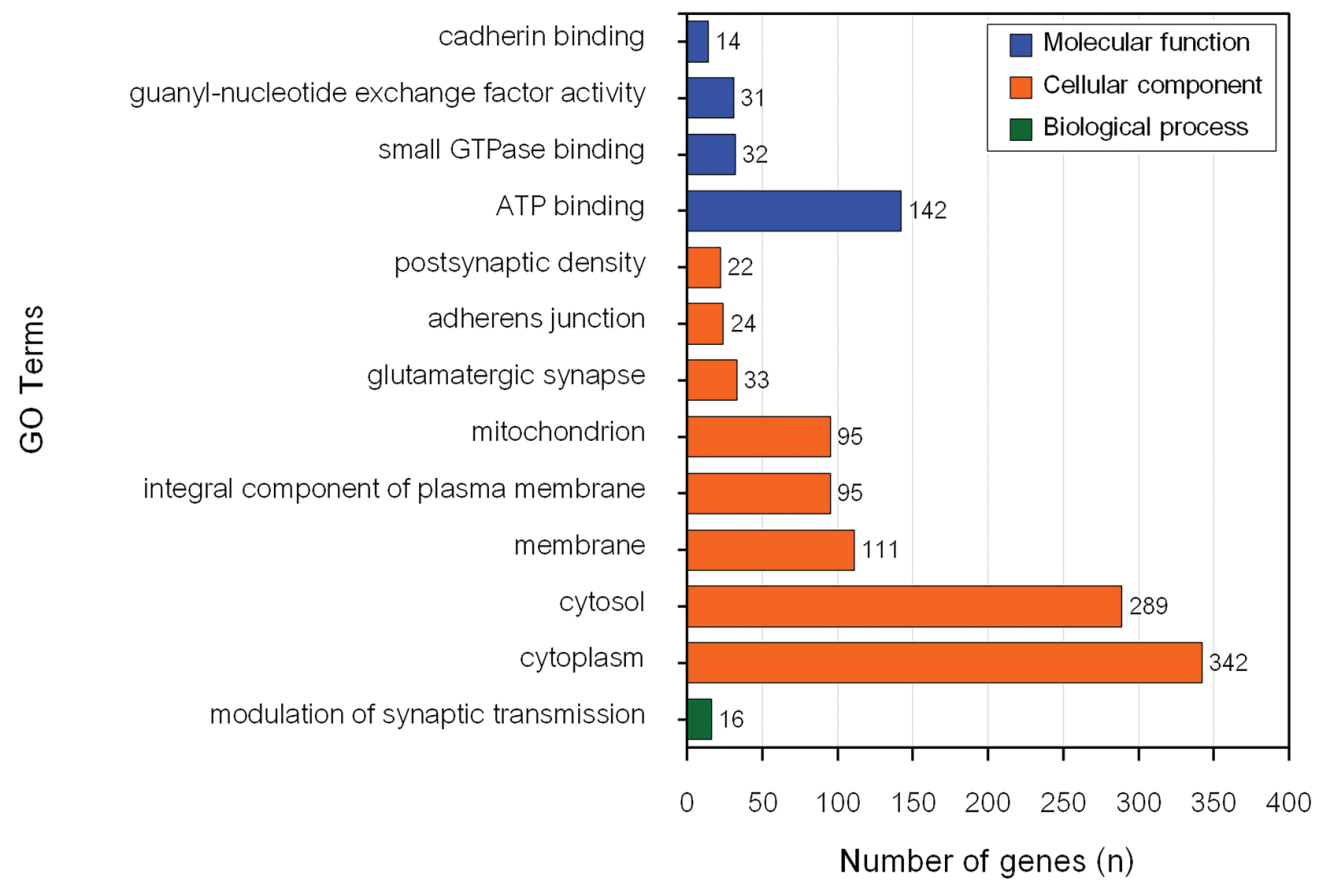
Functional terms significantly enriched corresponding to genes associated with fat yield.

The GO enrichment and KEGG pathway analyses of genes associated with MY revealed 26 significant BP, CC, and MF GO terms (Figure 2) and 1 significant KEEG pathway (Table 2). Conversely, there were 13 significant BP, CC, and MF GO terms (Figure 3) and 16 KEGG pathways (Table 2) associated with genes influencing FY.

The most significantly enriched BP for MY candidate genes (p = 0.005) was potassium ion transmembrane transport (GO:0071805). However, nervous system development (GO:0007399) had the highest number of genes (30) in the input data set. Cytosol (GO:0005829) was the most significantly enriched CC for MY candidate genes (p = 1.9×10^−9^) and had the highest number of genes (339) from the input data set. ATP binding (GO:0005524) was the most significantly enriched MF for MY candidate genes (p = 3.1×10^−4^). Metal ion binding (GO:0046872) had the highest number of genes (172) from the input data set for MY. The only significant pathway associated with MY was glutamatergic synapse (bta04724).

Modulation of synaptic transmission (GO:0071805) was the only significantly enriched BP for FY. Cytosol was the most significantly enriched CC for FY candidate genes (p = 7.1×10^−7^). Cytoplasm (GO:0005737) had the highest number of genes (342) for CC from the input data set. ATP binding (GO:0005524) was the most significantly enriched MF for FY candidate genes (p = 0.02) and had the highest number of genes (142) from the input data set. Focal adhesion was the most significantly enriched pathway for FY candidate genes (p = 0.004) and had the highest number of genes (34) from the input data set. In addition, seven significant GO terms, namely cytosol (GO:0005829), glutamatergic synapse (GO:0098978), adherens junction (GO:0005912), postsynaptic density (GO:0014069), ATP binding (GO:0005524), guanyl-nucleotide exchange factor activity (GO:0005085) and cadherin binding (GO:0045296) and glutamatergic synapse pathway (bta04724) were associated with both MY and FY in this population. Five of these significant GO terms (cytosol, adherens junction, postsynaptic density, ATP binding, and guanyl-nucleotide exchange factor activity) were previously reported to be associated with economically important traits in dairy cattle.

The CC cytosol (GO:0005829) is the part of the cytoplasm that contains particulate matter such as protein complexes that play an essential role in various cellular processes (https://www.ebi.ac.uk/QuickGO/term/GO:0005829). Several studies have indicated that genes involved in cytosol were associated with mastitis in dairy cows [33–35]. The adherens junction CC are essential for tissue morphogenesis, embryonic development, and maintenance of tissue architecture in adult organisms (https://www.ebi.ac.uk/QuickGO/term/GO:0034332). The adherens junction was previously reported to be associated with pregnancy loss in US Holstein cattle [36]. The ATP binding GO term plays a central role in energy transfer, enzyme catalysis, signal transduction, cellular movement, and DNA processes (https://www.ebi.ac.uk/QuickGO/term/GO:0005524). ATP binding was also reported to be associated with mastitis in Polish Holstein-Friesian cattle [37] and physical response to heat stress in Australian Holstein-Friesian [38] and Chinese Holstein cattle [39].

Guanyl-nucleotide exchange factor activity (GEF) refers to the ability of a protein to catalyze the exchange of guanosine diphosphate (GDP) for guanosine triphosphate (GTP) on small GTPases. GTPases are a class of enzymes involved in intracellular signaling and regulation of various cellular processes, including cell growth, differentiation, and vesicular transport (https://www.ebi.ac.uk/QuickGO/term/GO:0005085). The GEF was reported to be associated with male fertility in Italian Brown Swiss cattle [40].

The significantly enriched KEGG pathway glutamatergic synapse (bta04724) for MY and FY was also found to be significantly enriched in a previous study in this multibreed dairy population [7]. Cheruiyot et al [41] found eight enriched genes in the glutamatergic synapse pathway associated with heat tolerance in Australian Holstein cattle. In a similar study, Zamorano-Algandar et al [42] indicated that the glutamatergic synapse pathway was associated with MY in US Holstein affected by heat stress.

Potassium ion transmembrane transport, involved in the movement of potassium ions (K^+^) across the cell membrane, was the most significant enriched BP for MY candidate genes (https://www.ebi.ac.uk/QuickGO/term/GO:0071805). This process is crucial for maintaining the proper balance of potassium ions inside and outside the cell, which is essential for various cellular functions, including nerve transmission, muscle contraction, and maintenance of the resting membrane potential. Furthermore, potassium is an essential micromineral component for milk production in lactating cows during heat stress because it can mitigate the negative impact of heat stress on overall cow health [43]. Potassium ion transmembrane transport was also found to be associated with pregnancy loss in Holsteins cows [36] and fertility traits in Holstein Friesian, Jersey, and Holstein Friesian×Jersey cows [44].

The nervous system development GO term had the highest number of genes from the input data set for MY. This GO term plays an important role in the process by which the nervous system, including the brain and spinal cord, forms and matures during embryonic and postnatal development (https://www.ebi.ac.uk/QuickGO/term/GO:0007399). The nervous system development GO term was associated with FY in Chinese Holstein cattle [45].

The metal ion binding GO term had the highest number of genes from the input data set for MY. It plays an essential role in various cellular processes and is involved in the structure, function, and regulation of numerous proteins and enzymes (https://www.ebi.ac.uk/QuickGO/term/GO:0046872). Chropra et al [46] investigated the milk proteome in Sahiwal cattle and found that the majority of proteins had a functional role in metal ion binding. In addition, metal ion binding was reported to be associated with mastitis in German Holstein Frisian cattle [33].

Modulation of synaptic transmission, a significantly enriched BP for FY candidate genes, plays a crucial role in neuronal signaling and information processing in the nervous system (https://www.ebi.ac.uk/QuickGO/term/GO:0050804). Modulation of synaptic transmission was associated with maternal calving ease in Holstein cows [47].

Cytoplasm had the highest number of genes from the input data set among significantly enriched CC. Cytoplasm is a dynamic and essential component of cells because it provides a medium for cellular activities, supports metabolism, facilitates protein synthesis, enables cellular movement, and acts as a hub for signaling and communication within the cell (https://www.ebi.ac.uk/QuickGO/term/GO:0005737). Cytoplasm was found to be associated with mastitis in dairy cows [34]. Lastly, focal adhesion was the most significantly enriched pathway for FY and had the highest number of genes from the input data set. This finding was consistent with a previous study in the Thai multibreed dairy cattle population [7].

### Quantitative trait loci annotation analysis

**Figure 4:**
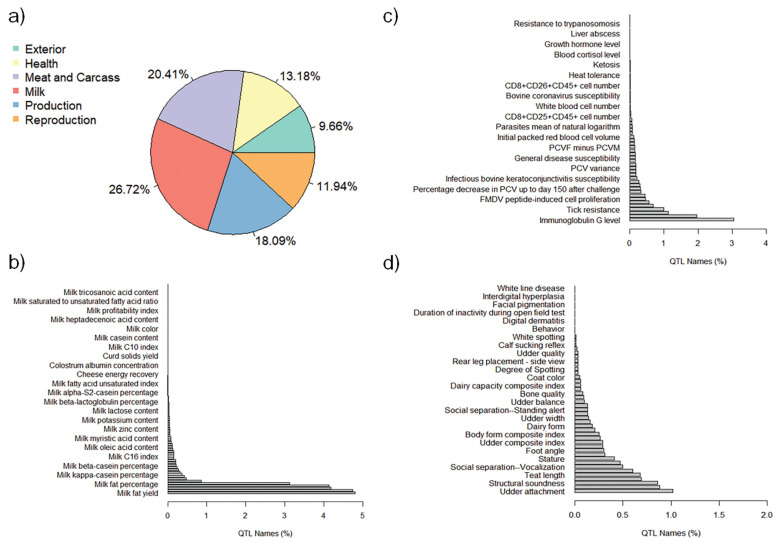
Percentages of total annotated QTL for MY by QTL class. a) Percentage of annotated QTL associated with different QTL classes; b) Percentage of annotated QTL associated with Milk trait category; c) Percentage of annotated QTL associated with Health trait category; and d) Percentage of annotated QTL associated with Exterior trait category. QTL, quantitative trait loci; MY, milk yield.

**Figure 5:**
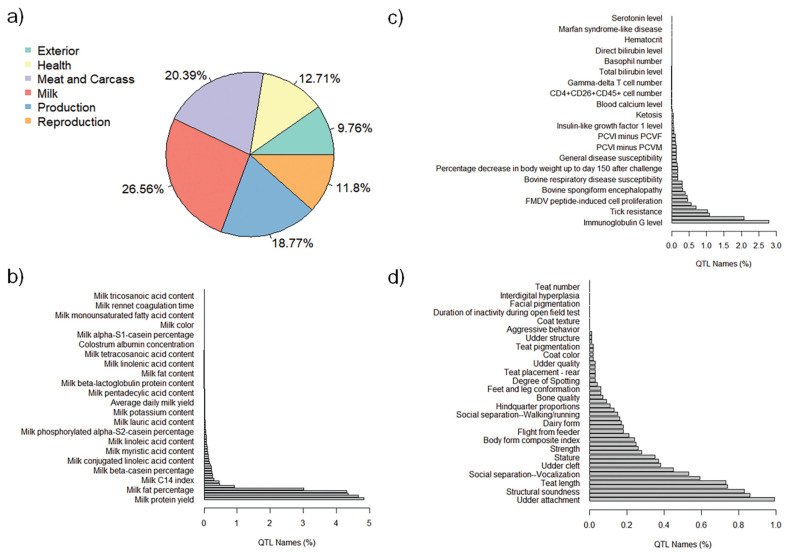
Percentages of total annotated QTL for FY by QTL class. a) Percentage of annotated QTL associated with different QTL classes; b) Percentage of annotated QTL associated with milk trait category; c) Percentage of annotated QTL associated with Health trait category; and d) Percentage of annotated QTL associated with Exterior trait category. QTL, quantitative trait loci; FY, fat yield.

The QTL annotation analysis identified a total of 9,334 QTL associated with MY and 8,977 QTL associated with FY based on SNP genotypes. These annotated QTL were distributed across all chromosomes, with chromosome 6 exhibiting the highest number of annotated QTL for both MY (885) and FY (1,487). Conversely, chromosome 28 had the lowest number of annotated QTL for MY (82), and chromosome 24 had the lowest number of annotated QTL for FY (87). The distribution of annotated QTL associated with MY and FY across different QTL classes is illustrated in Figure 4a and Figure 5a, respectively.

The analysis of annotated QTL revealed that approximately 27% of the QTL associated with MY and FY in the Thai multibreed dairy population belonged to the milk class. Other classes with sizeable QTL percentages were meat and carcass (20.41% for MY and 20.39% for FY), production (18.09% for MY and 18.77% for FY), health (13.18% for MY and 12.71% for FY), reproduction (11.94% for MY and 11.8% for FY), and exterior (9.66% for MY and 9.76% for FY). Further examination of the QTL associated with MY and FY within the milk class (Figure 4b and Figure 5b) revealed that a substantial portion of these QTL were related to specific milk traits. These included QTL associated with milk fat yield (4.80% for MY and 4.63% for FY), milk protein yield (4.74% for MY and 4.79% for FY), milk protein percentage (4.18% for MY and 4.31% for FY), and milk yield (4.13% for MY and 4.28% for FY). These percentages highlight the importance of milk-related QTL in determining the genetic variation for MY and FY in this population.

Interestingly, a relatively lower proportion of QTL within the health class (Figure 4c for MY and Figure 5c for FY) and exterior class (Figure 4d for MY and Figure 5d for FY) were associated with adaptation traits relevant to the tropical environment in Thailand. These traits included heat tolerance, respiratory rate, tick resistance, resistance to trypanosomiasis, and coat color. This suggests that the genetic basis for adaptation traits in the Thai multibreed dairy population may be governed by a different set of genes or genomic regions.

There were 17 QTL in the health and exterior QTL classes for MY. These QTL were associated with 9 distinct genes: short chain dehydrogenase/reductase family 16C member 5 (*SDR16C5*), regulatory factor X4 (*RFX4*), myosin IA (*MYO1A*), phosphodiesterase 3A (*PDE3A*), RNU1-1 (*U1*), PTPRF interacting protein alpha 2 (*PPFIA2*), CD83 molecule (*CD83*), rho GTPase activating protein 15 (*ARHGAP15*), and catenin alpha 2 (*CTNNA2*). These genes have been consistently associated with physiological traits related to tropical adaptation across various dairy and beef cattle breeds [48–61]. Two of the identified genes, *ARHGAP15* and *CTNNA2*, have been identified as candidate genes under positive selection for tolerance to trypanosomiasis in Boran and N'Dama cattle [48, 49]. The *ARHGAP15* gene, encoding a protein that acts as a negative regulator of *RAC1*, a key mediator in the mitogen-activated protein kinase (MAPK) pathway, plays a critical role in regulating the immune response against intracellular parasites [49]. Conversely, the *CTNNA2* gene, a protein-coding gene, is involved in the development of the nervous system and has shown associations with climate adaptation in Mediterranean cattle [50] and signals of positive selection in Gir cattle populations [51].

Other genes of interest are *RFX4*, *MYO1A*, *PDE3A*, *U1*, and *PPFIA2*. The *RFX4* gene, a member of the *RFX* family of transcriptional regulators, influences the expression of major histocompatibility complex (*MHC*) class II genes and plays a significant role in brain development [52]. It may also be implicated in the environmental adaptation of Chinese indigenous cattle. The *MYO1A* gene, a candidate gene from the myosin superfamily associated with skin pigmentation in cattle [53] was reported to be associated with thermotolerance in Chinese indigenous cattle [54,55]. The *PDE3A*, *U1*, and *PPFIA2* genes were found to be associated with physiological indicators of heat stress in Holstein, including rectal temperature, respiration rate score, and drooling score [56,57]. These associations highlight the importance of these genes for cattle adaptability to heat stress conditions.

Gene *SDR16C5* is a member of the short-chain alcohol dehydrogenase/reductase superfamily of proteins. It plays a crucial role in the oxidation of retinol to retinaldehyde, an essential step in the conversion of retinol into retinoic acid. This process is related to ectopic melanocyte stem cell differentiation in the hair follicle niche [58]. *SDR16C5* was reported to be associated with coat color in various cattle breeds, including Angus, Charolais, Limousin, and Holstein [59]. These findings emphasize the role of *SDR16C5* in both pigment-related and adaptation traits. The *CD83* gene, a member of the immunoglobulin superfamily of receptors, plays a regulatory role in lymphocyte maturation, activation, homeostasis, and antibody response to immunization and infection [60]. Gene *CD83* plays an essential role in the initiation and regulation of innate and adaptive immune responses. It has also been proposed as a candidate gene for tick resistance in *Bos taurus* × *Bos indicus* crossbred cattle [61]. These outcomes underscore the significance of *CD83* in immune response mechanisms and its potential role in tick resistance.

This study revealed a significant number of QTL associated with MY and FY based on SNP genotypes. The annotated QTL were distributed across all chromosomes, with chromosome 6 exhibiting the highest number of QTL for both MY and FY. This study also found that a substantial proportion of the annotated QTL were associated with the milk class, indicating its importance in the Thai multibreed dairy cattle population. This finding agreed with previous research conducted with Holstein and Holstein crossbred cattle.

This study also identified specific genes associated with MY within the health and exterior QTL classes. Nine different genes, namely *SDR16C5*, *RFX4*, *MYO1A*, *PDE3A*, *U1*, *PPFIA2*, *CD83*, *ARHGAP15*, and *CTNNA2*, were found to be associated with physiological characteristics related to tropical adaptation in various dairy and beef cattle breeds. Among these genes, *ARHGAP15* and *CTNNA2* were reported to be candidate genes under positive selection for tolerance to trypanosomiasis in specific cattle populations. The *ARHGAP15* gene was found to play a role in the negative regulation of *RAC1*, a key mediator in the MAPK pathway involved in immune response regulation against intracellular parasites. The *CTNNA2* gene was linked to the development of the nervous system, neurological diseases, and climate adaptation in cattle populations.

The *RFX4* gene was implicated in the environmental adaptation of Chinese indigenous cattle, whereas the *MYO1A* gene was associated with skin pigmentation and thermotolerance in Chinese indigenous cattle. Three other genes, *PDE3A*, *U1*, and *PPFIA2*, were reported to be associated with physiological indicators of heat stress response in Holstein cattle, particularly rectal temperature. The *SDR16C5* gene, apart from its involvement in the oxidation of retinol, was linked to coat color variations in multiple cattle breeds. Lastly, the *CD83* gene, belonging to the immunoglobulin superfamily of receptors, was found to regulate lymphocyte maturation, activation, homeostasis, and immune response. Its potential role as a candidate gene for tick resistance was also highlighted. Overall, this study successfully combined multi-omics approaches to identify sets of genes associated with MY and FY in a Thai multibreed dairy cattle population, shedding light on the genetic factors underlying adaptation and milk production traits in cattle, and providing valuable insights for the improvement of genetic selection strategies and breeding programs in the Thai multibreed dairy cattle population.

## CONCLUSION

This study identified a set of genes associated with 305-day MY and FY that also contribute to the adaptability of Thai multibreed dairy cattle to tropical environmental conditions using comprehensive GWAS and QTL analyses. Genes *ARHGAP15*, *CTNNA2*, *RFX4*, *MYO1A*, and *CD83* are associated with disease resistance, environmental adaptation, skin pigmentation, and immune response. These genes may play crucial roles in enhancing the ability of dairy cattle to withstand tropical environmental challenges. Additionally, genes *PDE3A*, *U1*, *PPFIA2*, and *SDR16C5*, linked to heat stress response and coat color variation, are essential for thermotolerance and environmental adaptation. Our findings shed light on the genetic factors influencing milk production and adaptability of dairy cattle under tropical conditions. An increased understanding of the physiological functions of these genes and their relationship to milk production and adaptability traits can facilitate the development of sustainable genetic selection strategies and breeding programs in tropical environments.

## Figures and Tables

**Table 1:** Significant single nucleotide polymorphisms for milk and fat yield

Trait	SNP	Chromosome	Location (bp)	Closed gene	Consequence	p-value	Additive genetic variance (%)
MY	Hapmap48544-BTA-98418	2	118,054,344	*FBXO36*	Intron variant	6.3068E-05	0.0277
	BovineHD0600014293	6	51,856,565	*-*	Intergenic variant	5.41113E-05	0.0295
	ARS-BFGL-NGS-66945	8	45,862,659	*PTAR1*	Intron variant	4.42661E-07	0.0419
	BovineHD1300022510	13	77,747,905	*B4GALT5*	Intron variant	6.95948E-06	0.0259
	BovineHD1500006727	15	25,465,697	*-*	Intergenic variant	1.75061E-05	0.0338
	BovineHD2600015217	26	47,463,279	*PTPRE*	Intron variant	2.27407E-05	0.0284
FY	BovineHD0600014293	6	51,856,565	*-*	Intergenic variant	1.43838E-05	0.0349
	BovineHD0800000801	8	2,302,545	*-*	Intergenic variant	9.88316E-05	0.0043
	ARS-BFGL-NGS-66945	8	45,862,659	*PTAR1*	Intron variant	5.24747E-05	0.0285
	BovineHD1500006717	15	25,398,861	-	Intergenic variant	3.33383E-05	0.0373

SNP, single nucleotide polymorphisms; MY, milk yield; FY, fat yield.

**Table 2:** Significant Kyoto encyclopedia of genes and genomes pathways for milk and fat yield

Trait	Terms	Description	Number of genes (n)	p-value	Genes
MY	bta04724	Glutamatergic synapse	26	0.00219	*GLS2, ITPR1, SLC1A2, GRIK4, CACNA1A, CACNA1D, ADCY1, CACNA1C, GRIK2, ADCY5, PPP3CA, GNG10, GRM5, GRM7, GNG7, GRM8, MAPK1, HOMER3, GRIA4, HOMER1, PRKCA, GRIN2B, GRIN1, PLCB4, SHANK3, SHANK2*
FY	bta04510	Focal adhesion	34	0.00457	*SHC3, ROCK2, PDGFB, LAMC1, PIK3CB, MYLK, MAPK8, RAP1A, CCND2, CCND1, ERBB2, MAPK1, FYN, PIP5K1B, PIP5K1C, PAK3, PAK2, PAK5, VASP, PPP1R12A, ITGA4, BAD, ACTN1, ITGA1, MAPK10, COL4A2, COL4A1, BCL2, COL6A3, GRB2, ITGA6, DOCK1, ITGA9*
	bta04520	Adherens junction	17	0.00688	*TCF7L1, CREBBP, ACTN1, PTPRM, SORBS1, TGFBR1, FER, PARD3, ERBB2, CTNNA1, SNAI1, SNAI2, MAPK1, CTNNA3, FYN, CTNNA2, SSX2IP*
	bta04071	Sphingolipid signaling pathway	23	0.00688	*CERS3, CERS4, CERS6, SGMS1, SPHK2, ROCK2, PRKCE, PPP2R3A, PIK3CB, PLD1, KNG1, GNAI1, MAPK10, PPP2CB, MAPK8, PPP2R2B, PPP2R5E, BDKRB2, BCL2, MAPK1, FYN, PLCB1, TP53*
	bta04724	Glutamatergic synapse	22	0.00688	*HOMER1, GLS2, GRIK3, SLC1A2, GRIK4, PLA2G4A, GRIK1, ADCY1, GRIK2, PLD1, GRIN2C, GRIN2B, GNAI1, PPP3CA, GNG10, GRM5, GRM8, MAPK1, PLCB1, PRKACB, SHANK2, GRIA4*
	bta04390	Hippo signaling pathway	26	0.01469	*YAP1, BMPR2, AFP, AREG, AMOT, STK3, PPP2CB, CCND2, CCND1, CTNNA1, TP53BP2, DVL3, CTNNA3, CTNNA2, AMH, TCF7L1, FZD4, WNT7A, TGFBR1, GDF7, BMP5, DLG2, FRMD6, PPP2R2B, PARD3, SNAI2*
	bta04072	Phospholipase D signaling pathway	25	0.01931	*ARF2, SHC3, DGKB, PDGFB, ADCY1, PIK3CB, PLD1, PIK3R6, PIK3R5, CYTH3, GRM5, GRM8, MAPK1, FYN, PIP5K1B, PIP5K1C, AVPR1B, SPHK2, PLA2G4A, DNM3, DGKQ, AGTR1, GRB2, PLCB1, DGKI*
	bta04530	Tight junction	27	0.02750	*ROCK2, PRKAG2, NEDD4L, ACTR3B, AMOT, PPP2CB, EPB41L4B, MAPK8, TUBA1A, RAP1A, CCND1, ERBB2, PRKACB, VASP, PRKCE, ACTN1, RDX, ARPC5, MAPK10, DLG2, CLDN14, PPP2R2B, PARD3, CLDN34, CLDN16, AMOTL1, TJP2*
	bta04070	Phosphatidylinositol signaling system	18	0.02750	*DGKB, IPMK, ITPK1, CALML4, PIK3CB, INPP4A, INPP4B, INPP5A, SYNJ1, IMPA1, DGKQ, PI4KA, PLCE1, PIP5K1B, PIP5K1C, PLCB1, PLCD4, DGKI*
	bta04810	Regulation of actin cytoskeleton	32	0.02750	*ROCK2, PDGFB, PIK3CB, C8B, IQGAP2, ACTR3B, KNG1, MYLK, C6, BDKRB2, MAPK1, PIP5K1B, PIP5K1C, PAK3, PAK2, FGF23, PAK5, ARHGEF12, PPP1R12A, ITGA4, ACTN1, ITGA1, RDX, ARPC5, DIAPH2, RGCC, DIAPH3, FGF19, ARHGEF4, ITGA6, DOCK1, ITGA9*
	bta04012	ErbB signaling pathway	16	0.03543	*SHC3, BAD, PIK3CB, AREG, MAPK10, MAPK8, NRG3, ERBB2, ABL1, ABL2, MAPK1, GRB2, PAK3, PAK2, CAMK2G, PAK5*
	bta01522	Endocrine resistance	17	0.03709	*JAG2, JAG1, SHC3, BAD, ADCY1, PIK3CB, ABCB11, ESR2, MAPK10, MAPK8, CCND1, ERBB2, BCL2, MAPK1, GRB2, TP53, PRKACB*
	bta04611	Platelet activation	20	0.03709	*VASP, FGB, FGA, PPP1R12A, ARHGEF12, ROCK2, PLA2G4A, ADCY1, PIK3CB, PIK3R6, GNAI1, MYLK, PIK3R5, RAP1A, TBXA2R, MAPK1, FYN, PLCB1, PRKACB, PRKG1*
	bta04360	Axon guidance	26	0.03709	*SEMA7A, BMPR2, ROCK2, SEMA3E, PIK3CB, GNAI1, ROBO1, PPP3CA, EFNB3, ABL1, MAPK1, FYN, PAK3, PAK2, CAMK2G, PAK5, EPHA5, ARHGEF12, EPHA6, UNC5D, GDF7, NFATC4, CDK5, PARD3, PLXNB1, EPHA3*
	bta04371	Apelin signaling pathway	22	0.03709	*HDAC4, RYR2, HDAC5, JAG1, SPHK2, PRKCE, PRKAG2, CALML4, ADCY1, PIK3R6, TGFBR1, GNAI1, MYLK, PIK3R5, MYL4, ADCY10, GNG10, CCND1, AGTR1, MAPK1, PLCB1, PRKACB*
	bta04144	Endocytosis	33	0.03709	*ARF2, VPS4A, AGAP1, NEDD4L, ASAP1, PLD1, ACTR3B, CYTH3, CAPZB, PSD4, GRK7, PSD3, PIP5K1B, PIP5K1C, LDLRAP1, CCR5, RAB11FIP3, WASHC1, EPS15, AP2M1, SNX5, ARFGEF2, SMURF1, ARPC5, AP2B1, TGFBR1, DNM3, ACAP2, PARD3, CAPZA2, AMPH, VPS45, SMAP1*
	bta00562	Inositol phosphate metabolism	14	0.04396	*IPMK, ITPK1, PIK3CB, INPP4A, INPP4B, INPP5A, SYNJ1, IMPA1, PI4KA, PLCE1, PIP5K1B, PIP5K1C, PLCB1, PLCD4*

KEGG, Kyoto encyclopedia of genes and genomes; MY, milk yield; FY, fat yield.

## SUPPLEMENTARY MATERIAL

**Supplementary Figure S1**. Variant effect predictors (VEP) annotations for the variants associated with milk yield.

**Supplementary Figure S2**. Variant effect predictors (VEP) annotations for the variants associated with milk yield.

**Supplementary Figure S3**. Manhattan plot of percentages of the additive genetic variance for milk yield and fat yield contributed by each SNP.

**Supplementary Table S1**. Number of genes per chromosome for milk yield (MY) and fat yield (FY) identified by SNPs genotypes inside or within 15 kb upstream and 15 kb downstream of genes in the Ensembl Bos taurus genome assembly
