# Supplementary material for: Genetic factors influencing milk and fat yields in tropically adapted dairy cattle: insights from quantitative trait loci analysis and gene associations
Source: Anim Biosci. 2023 Nov 1;37(4):576–90. doi: 10.5713/ab.23.0246 (PMC10915225; doi:10.5713/ab.23.0246)
Supplement: Supplementary file 2 [file ab-23-0246-Supplementary-Fig-S2.pdf]

Genetic factors influencing milk and fat yields in tropically adapted dairy cattle: insights from quantitative trait loci analysis and gene associations

Thawee Laodim[1,2], Skorn Koonawootrittriron[2,3,*], Mauricio A. Elzo[2,4], Thanathip Suwanasopee[2,3], Danai Jattawa[2,3], and Mattaneeya Sarakul[2,5]

*** Corresponding author: Skorn Koonawootrittriron**

Tel: +66-2-579-1120 Ext. 17, Fax: +66-2-579-1120, E-mail: agrskk@ku.ac.th

[1] Department of Animal Science, Faculty of Agriculture at Kamphaeng Saen, Kasetsart University Kamphaeng Saen Campus, Nakhon Pathom, 73140, Thailand

[2] Tropical Animal Genetic Special Research Unit (TAGU), Kasetsart University, Bangkok, 10900, Thailand

[3] Department of Animal Science, Faculty of Agriculture, Kasetsart University, Bangkok, 10900, Thailand

[4] Department of Animal Sciences, University of Florida, Gainesville, 32611-0910, FL, USA

[5] Department of Animal Science, Faculty of Agriculture and Technology, Nakhon Phanom University, Nakhon Phanom, 48000, Thailand

ORCID

Thawee Laodim	https://orcid.org/0000-0003-1993-0454
Skorn Koonawootrittriron	https://orcid.org/0000-0001-6170-7876
Mauricio A. Elzo	https://orcid.org/0000-0002-9319-3846
Thanathip Suwanasopee	https://orcid.org/0000-0002-9707-6428
Danai Jattawa	https://orcid.org/0000-0003-1398-0282
Mattaneeya Sarakul	https://orcid.org/0000-0003-4041-7938

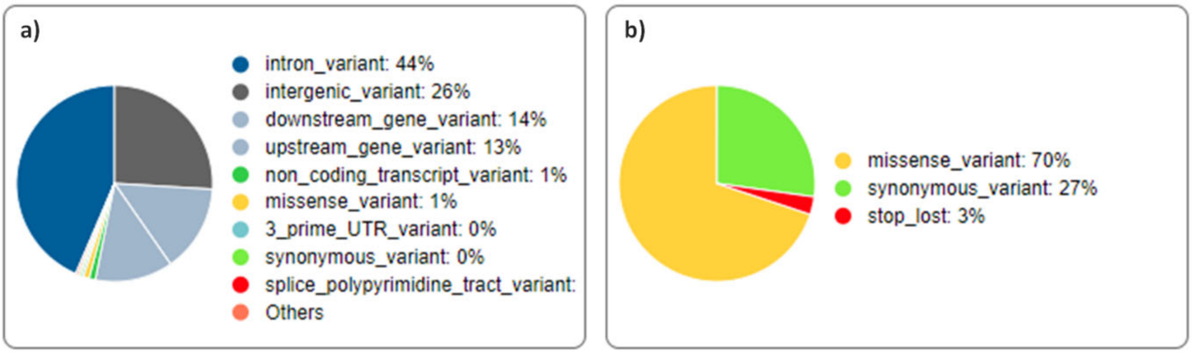

Supplemental Figure S1. Variant effect predictors (VEP) annotations for the variants associated with milk yield. a) Overall distribution of VEP annotations for all variants within 15 kb upstream to 15kb downstream of the reference bovine genome assembly. b) Annotation distribution for the variants present in coding sequences of all variants.

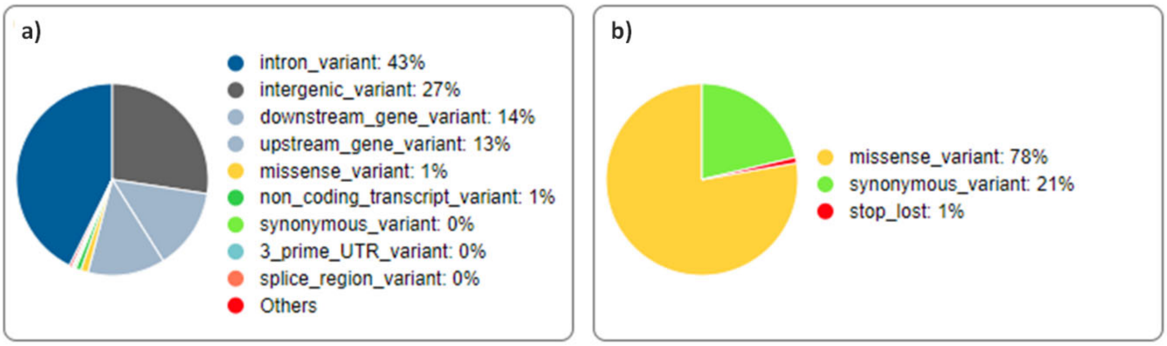

Supplemental Figure S2. Variant effect predictors (VEP) annotations for the variants associated with milk yield. a) Overall distribution of VEP annotations for all variants within 15 kb upstream to 15kb downstream of the reference bovine genome assembly. b) Annotation distribution for the variants present in coding sequences of all variants.
