# Supplementary material for: Genetic factors influencing milk and fat yields in tropically adapted dairy cattle: insights from quantitative trait loci analysis and gene associations
Source: Anim Biosci. 2023 Nov 1;37(4):576–90. doi: 10.5713/ab.23.0246 (PMC10915225; doi:10.5713/ab.23.0246)
Supplement: Supplementary file 3 [file ab-23-0246-Supplementary-Fig-S3.pdf]

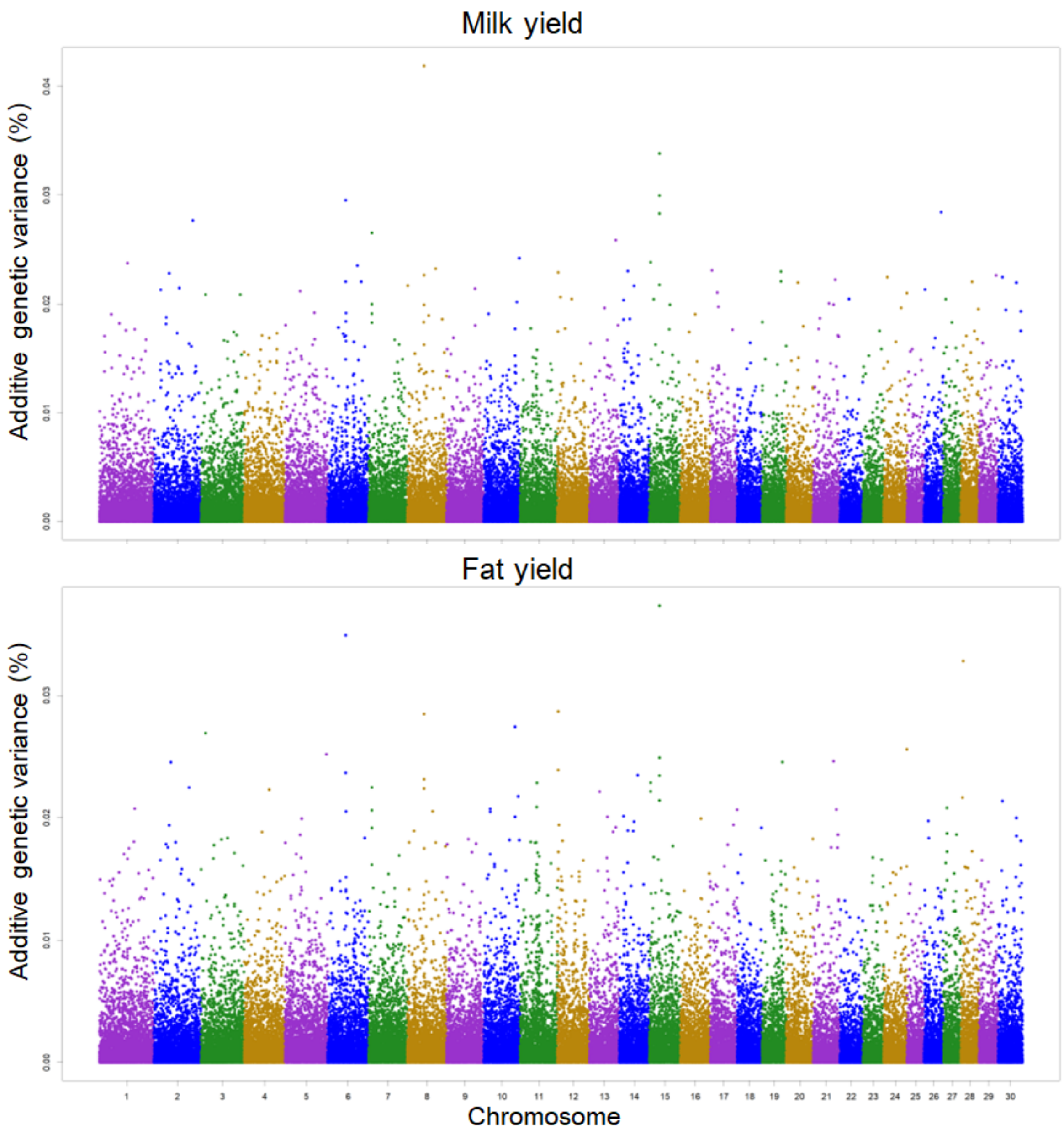

Supplemental Figure S3. Manhattan plot of percentages of the additive genetic variance for milk yield and fat yield contributed by each SNP.
