# Supplementary material for: Genetic factors influencing milk and fat yields in tropically adapted dairy cattle: insights from quantitative trait loci analysis and gene associations
Source: Anim Biosci. 2023 Nov 1;37(4):576–90. doi: 10.5713/ab.23.0246 (PMC10915225; doi:10.5713/ab.23.0246)
Supplement: Supplementary file 4 [file ab-23-0246-Supplementary-Table-S1.pdf]

Supplementary Table S1. Number of genes per chromosome for milk yield (MY) and fat yield (FY) identified by SNPs genotypes inside or within 15 kb upstream and 15kb downstream of genes in the Ensembl *Bos taurus* genome assembly

Chromosome	Number of genes (n)	
	MY	FY
1	95	80
2	112	88
3	125	85
4	84	80
5	116	86
6	63	56
7	91	80
8	57	49
9	54	41
10	85	86
11	92	75
12	30	38
13	51	62
14	47	29
15	93	81
16	61	46
17	59	51
18	79	63
19	87	79
20	42	38

21	60	48
22	50	48
23	86	44
24	34	33
25	54	48
26	31	31
27	19	19
28	39	47
29	31	41
X	24	32
